# Knowledge Gaps and Educational Interventions in Dietary Supplement Regulations Among Resident Physicians

**DOI:** 10.7759/cureus.79425

**Published:** 2025-02-21

**Authors:** Loai Azar, Margaret Neola, Jennifer Tran, Serenity Budd

**Affiliations:** 1 Internal Medicine, Banner - University Medical Center Tucson, Tucson, USA; 2 Nutrition, MedStar Washington Hospital Center, Washington DC, USA; 3 Internal Medicine, MedStar Washington Hospital Center, Washington DC, USA; 4 Biostatistics and Epidemiology, MedStar Washington Hospital Center, Washington DC, USA

**Keywords:** dietary supplements, fda, internal medicine, knowledge, physicians, regulations, residents

## Abstract

Background: Dietary supplement usage has significantly increased in the United States, even though dietary supplements lack premarketing Food and Drug Administration (FDA) approval and are susceptible to adulteration and contamination. This, in addition to a historical lack of standardized educational content for physicians on dietary supplements, poses major safety concerns.

Objective: This study aims to evaluate internal resident physicians’ knowledge of dietary supplement regulations with and without the provision of educational material.

Methods: A quasi-experimental quality improvement study was conducted at a community-based academic tertiary care hospital. Surveys were distributed to all residents to evaluate their knowledge of dietary supplement regulations with and without the provision of educational materials.

Results: Out of 100 possible participants, the initial survey had a 56% response rate, with a 42.8% average knowledge test score. Notably, 75% of participants correctly identified that dietary supplements do not require FDA approval before marketing. However, knowledge gaps were evident in efficacy, safety, adverse event reporting, and third-party certification awareness. There were no significant differences in knowledge based on gender, post-graduate year, or educational track. The post-intervention survey, with a 29% response rate, demonstrated significant improvements in knowledge across all tested aspects of FDA regulations, with an average test score of 75%.

Conclusion: The study found a knowledge gap among resident physicians regarding FDA regulations of dietary supplements that can be addressed with short and structured educational material.

## Introduction

Dietary supplement use has been increasingly popular in the United States (US). A dietary supplement is defined as a product taken by mouth, other than conventional food, that contains one or more dietary ingredients such as vitamins, minerals, herbs, other botanicals, or amino acids [1]. Consumer surveys have demonstrated that dietary supplement use has increased by 8-10% within the last 10 years, encompassing around 77% of the US population [2,3]. Healthcare professionals recommended less than a quarter of supplement use, and most supplement users stated their use was due to personal choice [3,4]. It is estimated that around 33% of supplement users do not discuss supplement intake with their healthcare providers, which raises multiple safety concerns [5,6]. Dietary supplements do not undergo premarketing Food and Drug Administration (FDA) approval under the Dietary Supplement Health and Education Act of 1994 (DSHEA), and the FDA has warned of possible adulteration and contamination of dietary supplements, which occurs when dietary supplements deliberately contain substances that are not labeled or unintentionally contain undisclosed substances due to the lack of quality control, respectively [7,8]. When dietary supplement use is discussed with patients, physicians often fail to discuss FDA regulations of dietary supplement use, including their effectiveness and potential risks from adulteration and contamination [9]. Further complicating this point, prior studies have demonstrated that 65% of physicians did not have a reliable source of information for discussing dietary supplements with their patients [10]. Also, dietary supplement intake has been shown to increase with age and other prescription medication intake, which may increase the risk of drug-supplement interactions [2,3]. Dietary supplements only undergo post-marketing surveillance by the FDA. Usually, multiple adverse events occur before a supplement is removed from the consumer market [1]. Studies have previously shown that physicians are unaware of FDA regulations for dietary supplements, which may aggravate these health concerns [10,11]. The low disclosure rate of dietary supplement use with healthcare professionals may also impose a challenge for researchers trying to draw associations between dietary supplement use and health outcomes. Without proper documentation, retrospective studies are difficult.

Geller et al. estimated that around 23,000 emergency department visits in the US each year are related to dietary supplement use, with adverse events from weight loss products being a common issue, making it especially concerning in today’s growing public interest and market for weight loss products [12]. This number may be even higher given the fact that dietary supplement use is underreported. A survey performed by Cellini et al. on US military physicians demonstrated that around 60% of participant physicians observed adverse events related to dietary supplement use, and of those, only 18% reported it, and the most reported reason for non-reporting was a lack of knowledge on how to report adverse events [10]. Similar results were obtained in 2007 when surveying physicians at internal medicine residency training programs. The results found that more than 30% of physicians were unaware of dietary supplement regulations, and two-thirds of physicians were unclear about reporting adverse events. Moreover, the answers were not significantly different among different years of training or practice [11]. This reflects a long-time issue of the lack of standardized educational content on dietary supplements and their use for physicians during graduate medical education training. Dietary supplements and related health concerns are often not included in the educational curriculum in medical schools or graduate medical education programs to this day. Therefore, we expect a continued trend that resident physicians, who later become independently practicing physicians, often do not discuss dietary supplement use, its risks, and its benefits with patients. This highlights an area for improvement that may increase patient safety and outcomes. Here, we performed a quality improvement study to evaluate internal medicine resident physicians' change in knowledge of FDA regulations related to dietary supplement use before and after an educational intervention.

## Materials and methods

Between January 2023 and May 2023, a quasi-experimental quality improvement study was conducted to evaluate and improve internal medicine resident physicians’ knowledge of FDA regulations of dietary supplements. Participants included all first-, second-, and third-year internal medicine resident physicians at MedStar Washington Hospital Center, USA. The participants were divided into two groups: a control group, where their knowledge was assessed using survey A (Appendix A), and an intervention group, where their knowledge was assessed using survey B (Appendix B) post-intervention. Surveys were administered via email using Microsoft Forms (Microsoft Corp., Redmond, WA, USA). The intervention was comprised of educational materials, including a pre-recorded PowerPoint presentation (Microsoft Corp., Redmond, WA, USA) and an informational brochure.

All 100 potential participants received an invitation to join the study by email, along with survey A. The educational intervention was implemented for all residents after more than 50% of participants completed the initial survey or after two months of survey distribution, whichever came first.

After reviewing the educational material, participants were asked to complete survey B. Only those who completed one of the educational materials were permitted to submit responses. Survey B closed once it achieved a response rate of over 50% or after two months of distributing the study materials, whichever came first. Responses were not tracked as the data was de-identified. Overlap between the intervention and control group participants was possible.

With permission, both surveys included four knowledge questions internally validated by Ashar et al. [11]. The fifth question was a direct yes and no question that did not require internal validation. Participants were provided with a five-dollar and a 10-dollar gift card, respectively, to complete the first and second surveys. Each survey was estimated to take 3-4 minutes to complete while reviewing any of the educational materials was estimated to take 10-15 minutes.

The educational materials focused on various objectives, including understanding the FDA's role in regulating dietary supplements, knowledge of quality assurance regulations, familiarity with safety and efficacy regulations, and knowledge of how to report adverse events to the FDA.

Demographic variables and study material choice were summarized using frequencies and percentages. Fisher’s exact test was used to test for differences in demographic characteristics between the intervention and control groups. The percentage correct for each question on the assessments by intervention versus control was shown in a bar chart. Chi-squared tests were used to test the difference in the proportion of correct answers for each of the five test questions between the intervention and the control group. A two-sample t-test was used to test the difference in mean scores between the intervention and the control group. All analyses were conducted in R version 4.3.1 (R Foundation for Statistical Computing, Vienna, Austria) with an alpha level of 0.05. This study protocol was approved by the Georgetown University/Medstar Health Review Board (Study ID: STUDY00005727).

We adhered to the Strengthening the Reporting of Observational Studies in Epidemiology (STROBE)-nut guidelines to ensure transparent and comprehensive reporting of our research methodology and findings [13].

## Results

The initial knowledge survey yielded a 56% response rate, equating to 56 participants being included in the control group. Thirty-four respondents (62%) were female, and 53 respondents (95%) were aged between 25 and 34. There were 26 (46%) respondents in postgraduate year 1 of training (PGY-1), 15 (27%) respondents in PGY-2, and 14 (25%) respondents in PGY-3. Eight (14.3%) participants were in the preliminary medicine educational tract, and all the preliminary intern physicians were in PGY-1 (Table 1).

**Table 1:** Demographic characteristics of the control and intervention groups ^1 ^indicates Fisher's exact test PGY: postgraduate year

Demographics	Overall N = 85	Control N = 56	Intervention N = 29	p-value^1^
Gender				0.7
Male	33 (40%)	20 (36%)	13 (46%)	
Female	49 (59%)	34 (62%)	15 (54%)	
Non-binary	1 (1%)	1 (2%)	0 (0%)	
Unknown	2	1	1	
PGY				>0.9
1	41 (48%)	26 (46%)	15 (52%)	
2	23 (27%)	15 (27%)	8 (28%)	
3	20 (24%)	14 (25%)	6 (21%)	
4	1 (1%)	1 (2%)	0 (0%)	
Educational tract				>0.9
Preliminary	12 (14%)	8 (14%)	4 (14%)	
Categorical	73 (86%)	48 (86%)	25 (86%)	
Age				>0.9
18-24	2 (2%)	1 (2%)	1 (3%)	
25-34	80 (94%)	53 (95%)	27 (93%)	
35-44	3 (4%)	2 (4%)	1 (3%)	

**Figure 1:**
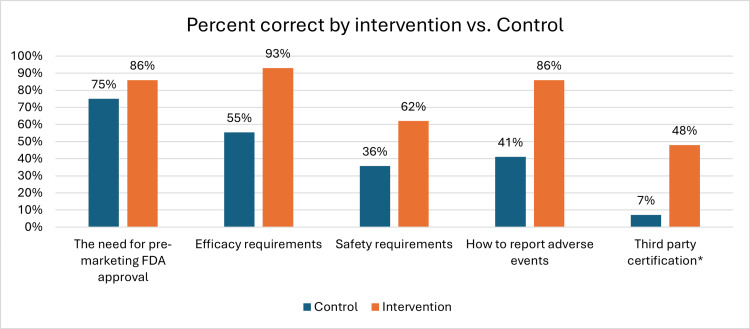
The average score in the intervention group (M = 75.17, SD = 27.07) was significantly higher than the average score in the control group (M = 42.86, SD = 24.25), t(83) = -5.60, p < 0.001 FDA: Food and Drug Administration *The fifth question indirectly reflects residents’ knowledge of quality and manufacturing regulations by asking about awareness of dietary supplement third-party certification

The average test score for dietary supplement knowledge questions was 42.86% pre-intervention. Forty-two participants (75%) correctly indicated that dietary supplements do not require FDA approval before marketing. Thirty-one (55%) and 20 (36%) participants, respectively, correctly identified that dietary supplements do not require proof of efficacy and safety before marketing. Thirty-three (59%) participants were unable to correctly identify how to report adverse events related to dietary supplement use and only four (7%) were aware of third-party certification for dietary supplements (Figure 1). There was no statistically significant difference in the average test score with subgroup analysis by PGY.

For the post-intervention knowledge survey, there were 29 participants with a 29% response rate after two months of distributing the educational material. Most people used either the brochure or both materials to study for the assessment. Only four people used just the PowerPoint (Table 2).

**Table 2:** Percentage of participants who used each or both educational materials

Intervention group	N = 291
Study material	
Both	12 (41%)
Brochure	13 (45%)
PowerPoint	4 (14%)

There was no statistically significant difference between the two samples’ demographics regarding gender, PGY, age, or educational tract. Post-intervention knowledge questions demonstrated improvement in all of the five tested aspects of FDA regulations of dietary supplements with an average test score of 75.17%. The only question that did not have a statistically significant improvement in knowledge from the pre- to post-intervention test was about the need for premarketing FDA approval of dietary supplements. However, the remaining four questions showed a statistically significant improvement in pre- and post-intervention knowledge (p < 0.05) (Figure 1).

## Discussion

This study assessed resident physicians’ knowledge of FDA regulations for dietary supplements with and without the completion of at least one of two educational materials to help improve their knowledge. In 2007, Ashar et al. assessed resident physicians’ knowledge of FDA regulations of dietary supplements at over 15 internal medicine residency programs across the country and found the average test score for the knowledge question to be 58.5% [11]. Our study demonstrated similar findings with Asher et al., as our average test score was 42.8% without intervention. However, it is concerning that despite the passage of a significant amount of time, the knowledge of dietary supplements has remained low amongst new physicians.

Studies have shown that less than 25% of physicians do not discuss dietary supplement use with their patients [9,10,14,15]. This could be due to a lack of knowledge of dietary supplements, as most physicians do not feel that they receive enough education about dietary supplements during medical school and post-graduate residency training [16,17]. Not only did this study demonstrate limited knowledge of the FDA regulations on dietary supplements, but it also demonstrated that there was no difference in knowledge between the different years of residency training, confirming the lack of a structured curriculum addressing this topic. This finding is unlikely to be limited to the study’s location, as the Accreditation Council for Graduate Medical Education (ACGME) internal medicine educational curriculum requirements do not contain dietary supplement education as a required component of the curriculum. Similarly, Ashar et al. found no difference in knowledge among different PGY across more than 15 residency programs [11].

Dietary supplement use in the US has risen significantly, increasing by more than 8% from 2007 to 2018, reaching almost 80% of the population [2,3]. As a consequence, the incidence of dietary supplement-related adverse events, such as drug-supplement interactions and other adverse events from dietary supplement contamination and adulteration, is also projected to increase. Adverse events from dietary supplement contamination and adulteration or drug-supplement interactions can be life-threatening, requiring emergency room visits and hospitalization [12,18]. However, physicians and healthcare workers have historically underreported adverse events associated with dietary supplement use [10,19]. This study reveals that resident physicians lack awareness of the existing reporting methods for adverse events related to dietary supplements, underscoring the pressing need to educate physicians on the importance of reporting and how to do it. This is imperative, as post-marketing surveillance through reporting is the primary method for removing harmful dietary supplements from the market, given the lack of pre-marketing safety studies.

Although this survey did not directly measure knowledge about the potential contamination or adulteration of dietary supplement products, it did ask about knowledge of third-party organizations that certify supplements and their ingredients. We found that most resident physicians are unaware of third-party certification (n = 52, 93%); however, this does not necessarily indicate that physicians are unaware of the risks of contamination and adulteration. Awareness of third-party certification could aid providers in providing better patient education and thus help patients select safe and effective supplements. This is very important as surveys have shown that more than 50% of dietary supplement users trust the labels on their products [20,21].

The study demonstrated statistically significant improvement in resident knowledge following the implementation of educational material. However, despite this improvement, the average test score remained low, possibly due to the digital nature of the intervention. In-person lectures may offer a more effective approach to improving dietary supplement knowledge as they allow for greater interactivity, enabling participants to ask questions directly. Furthermore, there is the possibility of selection bias and overlap between the intervention and control groups, as participants had the option to complete the first assessment, the second assessment, or both. For instance, individuals who were more eager to learn or confident in their abilities might have been more inclined to complete the second assessment, potentially inflating the intervention group's score. Another limitation of our study is we could not evaluate which study material led to better knowledge outcomes, as most participants opted to complete the educational brochure instead of viewing the pre-recorded PowerPoint slides.

The simplicity of the study design and the use of internally validated knowledge questions, including four out of five questions from a study published by Dr. Ashar in 2007 [11], are the key strengths of this study. The fifth question, which indirectly assessed participants' knowledge of quality and manufacturing regulation by inquiring about their awareness of a third-party certification organization for dietary supplements, was not previously validated. It is worth mentioning that FDA regulations governing dietary supplements have remained unchanged since 2007 and that legislation to require independent third-party certification for products sold in the US could be another positive solution to helping reduce adverse outcomes [8].

Medical learning can often be self-directed, and this study did not explore the reasons for the resident physicians' knowledge deficit. It is possible that factors beyond the educational curriculum, such as a lack of interest in the subject or a lack of awareness of its significance, may have contributed to this knowledge deficit and should be evaluated in future studies.

This emphasizes the need for more comprehensive education on dietary supplements during residency programs and medical school training, as it appears that the current curriculum is not providing sufficient coverage on this important topic. As shown in other studies, educating physicians about FDA regulations of dietary supplements can help guide patient discussions about supplement use, improve patient knowledge of supplements, and lead to safer consumption and fewer adverse events [20,21].

## Conclusions

The study found a significant knowledge gap among resident physicians regarding FDA regulations on dietary supplements, emphasizing the critical need for comprehensive education on this topic during medical school and residency programs. Addressing this gap through structured and interactive learning interventions, such as in-person lectures or mandatory curriculum modules, could better prepare physicians to counsel patients effectively on dietary supplement use. Enhanced physician knowledge has the potential to reduce adverse events, improve patient safety, and ensure more informed discussions about the risks and benefits of dietary supplements in clinical practice.

## Appendices

Appendix A 

Residents Knowledge in Dietary Supplements Regulations (Part 1)

**Table 3:** Survey A FDA: Food and Drug Administration; PGY: postgraduate year

Question	Answer options
Do you consent to participate in this survey?	Yes
	If not, please close this page
What is your gender?	Female
	Male
	Non-binary
	Prefer not to say
What year of residency are you in?	PGY1
	PGY2
	PGY3
	Other
How old are you?	18-24
	25-34
	35-44
	45-54
Which of the following statements regarding government approval is correct?	Orlistat did not require approval by the FDA prior to marketing, while Blubbermelt XL did
	Neither orlistat nor Blubbermelt XL required approval by the FDA prior to marketing
	Blubbermelt XL did not require approval by the FDA prior to marketing, while orlistat did
	Both orlistat and Blubbermelt XL required approval by the FDA prior to marketing
Which one of the following statements is correct regarding efficacy data?	Manufacturers of Blubbermelt XL are required to show only limited efficacy data to the FDA prior to being marketed, while manufacturers of orlistat are required to provide much more.
	Manufacturers of orlistat are required to show only limited efficacy data to the FDA prior to being marketed, while manufacturers of Blubbermelt XL are required to provide much more.
	Manufacturers of Blubbermelt XL and orlistat are required to show equivalent amounts of efficacy data to the FDA prior to being marketed.
	Manufacturers of Blubbermelt XL are not required to show any efficacy data to the FDA prior to being marketed, while manufacturers of orlistat are.
Which of the following statements regarding safety issues is correct?	Manufacturers of Blubbermelt XL are not required to show any safety data to the FDA prior to being marketed, while manufacturers of orlistat are.
	Manufacturers of Blubbermelt XL are required to show only limited safety data to the FDA prior to being marketed, while manufacturers of orlistat are required to provide much more.
	Manufacturers of orlistat are required to show only limited safety data to the FDA prior to being marketed, while manufacturers of Blubbermelt XL are required to provide much more. Manufacturers of Blubbermelt XL and orlistat are required to show equivalent amounts of safety data to the FDA prior to being marketed.
Which of the following statements regarding adverse event reporting is correct?	It is the professional duty of physicians to report the event to the American Dietary Supplement Council hotline.
	It is the professional duty of physicians to report the event to the retailer that sold the product, since they are legally required to disclose these events to the FDA.
	It is the professional duty of physicians to simply document the potential adverse event in the chart, since there is currently no adverse event reporting system for dietary supplements.
	It is the professional duty of physicians to report the event through the FDA's Medwatch system.
Have you ever heard about dietary supplement third-party certifications?	No
	Other, please specify:

Appendix B

Residents Knowledge in Dietary Supplements Regulations (Part 2)

Please fill this form after completing the study material.

**Table 4:** Survey B FDA: Food and Drug Administration; PGY: postgraduate year

Question	Answer options
Do you consent to participate in this survey?	Yes
	If not, please close this page
What study material did you use?	Brochure
	PowerPoint
	Both
What is your gender?	Female
	Male
	Non-binary
	Prefer not to say
What year of residency are you in?	PGY1
	PGY2
	PGY3
	Other
Are you preliminary or categorical?	Preliminary
	Categorical
How old are you?	18-24
	25-34
	35-44
	45-54
Which of the following statements regarding FDA approval is correct?	Tamsulosin, but not FIoGo, requires approval by the FDA prior to marketing.
	Neither tamsulosin nor FIoGo requires approval by the FDA prior to marketing by their manufacturers.
	Both Tamsulosin and FIoGo require approval by the FDA prior to marketing by other manufacturers.
	FIoGo, but not tamsulosin, requires approval by the FDA prior to marketing.
Which statement on efficacy data is true?	Manufacturers of tamsulosin are required to show limited efficacy data to the FDA, while manufacturers of FIoGo are required to show extensive efficacy data to the FDA prior to approval.
	Manufacturers of tamsulosin are required to show efficacy data to the FDA prior to approval, while manufacturers of FIoGo are not.
	Both manufacturers of FIoGo and tamsulosin are required to present equal amounts of efficacy data to the FDA prior to approval.
	Manufacturers of FIoGo are required to show efficacy data to the FDA prior to approval, while manufacturers of tamsulosin are not.
Which statement regarding safety regulations is correct?	Manufacturers of both tamsulosin and FIoGo are required to show equal amounts of safety data to the FDA prior to approval.
	Safety data is not required for either tamsulosin or FIoGo for FDA approval.
	Limited safety data is required on FIoGo prior to FDA approval, while extensive safety data is required on tamsulosin.
	Safety data is required on Tamsulosin prior to FDA approval, but it is not required on FIoGo.
What should be done in case of an adverse event with FIoGo?	Since you warned Mr. D about the risks of FIoGo and documented this, your professional duties have been fulfilled, as there is no adverse event reporting system for dietary supplements.
	It is your professional duty to tell the patient to report this to the retailer who sold the product.
	Reporting of adverse events for dietary supplements can be done the same way as that for prescription drugs; it is your professional duty to report this reaction through the FDA's Medwatch system.
	The American Dietary Supplement Council hotline is the appropriate mechanism for reporting this event; it is your professional duty to contact them.
Have you ever heard about third-party certifications for dietary supplements?	No
	Other
